# Deliberately infecting healthy volunteers with malaria parasites: Perceptions and experiences of participants and other stakeholders in a Kenyan‐based malaria infection study

**DOI:** 10.1111/bioe.12781

**Published:** 2020-07-09

**Authors:** Irene Jao, Vicki Marsh, Primus Che Chi, Melissa Kapulu, Mainga Hamaluba, Sassy Molyneux, Philip Bejon, Dorcas Kamuya

**Affiliations:** ^1^ KEMRI‐Wellcome Trust Research Programme Kilifi Kenya; ^2^ Centre for Tropical Medicine and Global Health Nuffield Department of Medicine University of Oxford Oxford UK

**Keywords:** Africa, challenge studies, controlled human infection studies, deliberate infection, developing countries, ethics

## Abstract

Controlled human malaria infection (CHMI) studies involve the deliberate infection of healthy volunteers with malaria parasites under controlled conditions to study immune responses and/or test drug or vaccine efficacy. An empirical ethics study was embedded in a CHMI study at a Kenyan research programme to explore stakeholders’ perceptions and experiences of deliberate infection and moral implications of these. Data for this qualitative study were collected through focus group discussions, in‐depth interviews and non‐participant observation. Sixty‐nine participants were involved, including CHMI study volunteers, community representatives and research staff. Data were managed using QSR Nvivo 10 and analysed using an inductive‐deductive approach, guided by ethics literature. CHMI volunteers had reasonable understanding of the study procedures. Decisions to join were influenced by study incentives, trust in the research institution, their assessment of associated burdens and motivation to support malaria vaccine development. However, deliberate malaria infection was a highly unusual research strategy for volunteers, community representatives and some study staff. Volunteers’ experiences of physical, emotional and social burdens or harms were often greater than anticipated initially, and fluctuated over time, related to specific procedures and events. Although unlikely to deter volunteers' participation in similar studies in furture, we argue that the dissonance between level of understanding of the burdens involved and actual experiences are morally relevant in relation to community engagement, informed consent processes, and ongoing support for volunteers and research staff. We further argue that ethics oversight of CHMI studies should take account of these issues in deciding whether consent, engagement and the balance of benefits and harms are reasonable in a given context.

## 1 INTRODUCTION

### 1.1 Controlled human malaria infection in endemic areas in low‐ to middle‐income countries (LMICs)

Controlled human malaria infection (CHMI) studies involve deliberately infecting healthy volunteers with malaria pathogens under controlled conditions to observe the natural history of the pathogen or to test drug or vaccine efficacy.^1^ Volunteers are then observed and monitored closely to facilitate a detailed evaluation of parasite growth dynamics and immunological responses to malaria infection.^2^ Deliberate infection allows the studying of early stages of infection which is otherwise difficult to assess, given that malaria infections are mostly asymptomatic at these early stages, adding to the social value of CHMI.^3^ Given these features, CHMI studies have increasingly been seen as an important scientific tool in malaria control and elimination.^4^

While controlled human infection (CHI) studies intrinsically carry different levels and forms of risk depending on the pathogen under study, many have had a good safety record due to the stringent research practices followed; studies are conducted under carefully controlled conditions where the type of pathogen, dosage, route and timing of administration are carefully planned to minimise probability of serious harm.^5^ Volunteers’ health and wellbeing are closely monitored by qualified clinical staff and volunteers are immediately treated should they become symptomatic or feel unwell.^6^ Additionally, pathogens used in CHI studies have been either self‐limiting or readily controlled with appropriate low‐risk treatments that are made immediately available to volunteers.^7^ Risks that CHI studies pose are further mitigated through health screening prior to enrolment.^8^ Where CHI studies have potential third‐party risks or safety issues, volunteers maybe be provided with prolonged residential or in‐patient accommodation.^9^

CHMI studies have been increasingly conducted in malaria‐endemic settings in LMICs in response to the need for more effective vaccines and therapies in areas where malaria is an important public health burden.^10^ A faster turn‐around of results and the involvement of smaller number of volunteers in CHI studies offer important advantages over traditional clinical trials.^11^ The social value of CHMI studies conducted in malaria endemic settings is argued on the basis of the high burden of malaria disease and deaths in these settings; relatively modest efficacy of recently developed malaria vaccines;^12^ and the view that CHMI studies may present less risk or harm to volunteers in these settings who have prior exposure to the infection, including through higher innate resistance to the pathogen as a result of naturally acquired or genetic resistance.^13^

### 1.2 Deliberate infection in controlled human infection studies

Deliberate infection of healthy volunteers is a core strategy underpinning CHI studies and their associated high social value. Deliberate infection serves to ensure standardisation of infection for the volunteers in a group/sub‐group, which allows researchers to study how different individuals respond to identical exposures and why their responses may differ.^14^ Not all CHI studies involving deliberate infection of healthy volunteers lead to progression to the disease; sub‐optimal pathogen levels may be used to study immune responses.^15^

Nonetheless, the intentional infection of healthy volunteers with pathogens raises a number of ethical and social issues. Taken overall, ethical issues for CHI studies have centred on concerns about the levels of harm or burdens that may be involved: how potential harms or burdens for individuals might be weighed up against the potential future social value of a study; issues around informed consent (particularly given risks of harm). Relatedly, concerns about undue inducement, given high levels of incentives or compensation that are often involved; and issues around public trust and reputational risks for the research enterprise as well as specific institutions.^16^

More specifically, there are concerns about whether intentionally infecting healthy volunteers is, in itself, ethically and morally permissible because there is no prospect of direct therapeutic benefits for volunteers.^17^ Hope and McMillan (2004) argue that the idea of inducing disease in a healthy individual is in itself not morally wrong since the aims of obtaining a new drug or vaccine are in line with the goals of medicine. At the same time, they note that: (i) for those unfamiliar with CHI study designs, there may be worries and hesitation to permit or participate in these studies, as they could be perceived as bearing considerable levels of risk; (ii) physicians may experience moral tensions if they feel their role of curing people is contrary to the potential for causing harm to healthy volunteers intentionally infected with a pathogen; and (iii) potential reputational harm towards healthcare systems may arise in populations with less exposure/understanding of research and/or where research and healthcare are offered in the same facilities by the same research/healthcare professional.^18^ Hope and McMillan further argue the importance of well‐considered and transparent guidelines and regulatory processes for CHI studies, in recognition of public expectations of medicine and medical research.

In this paper, we focus on a set of ethical issues related to the fundamental concept of ‘deliberate infection’, aiming to feed into ethics debates around the acceptability of deliberate infection and associated generation of harms. We draw on empirical ethics research embedded in a malaria CHMI study in a Kenyan research programme, adding to published findings in relation to general perceptions and experiences of the research approach.^19^ Empirical ethics studies are gaining popularity in bioethics, given their capacity to allow researchers to explore social realities of people affected by a phenomenon under study and the potential to inform normative debates by providing practical solutions.^20^ While the ethical dimensions of CHI study participants’ experiences in the USA have recently been described,^21^ the ethical issues surrounding the concept of deliberate infection has not, to our knowledge, been examined from the perspective of CHMI volunteers and research stakeholders in an LMIC.

## 2 METHODS

### 2.1 The study site and ‘parent’ CHMI study in Kenya

The Kenya Medical Research Institute (KEMRI)‐Wellcome Trust Research Programme (KWTRP) is a long standing international collaborative research programme with its headquarters in Kilifi County in coastal Kenya, a largely rural county where a majority of the population work in the informal sector or in subsistence farming. An increasingly urban population is emerging, including from recently established county government offices as part of a national devolution process and the expansion of a local university.^22^

**FIGURE 1:**
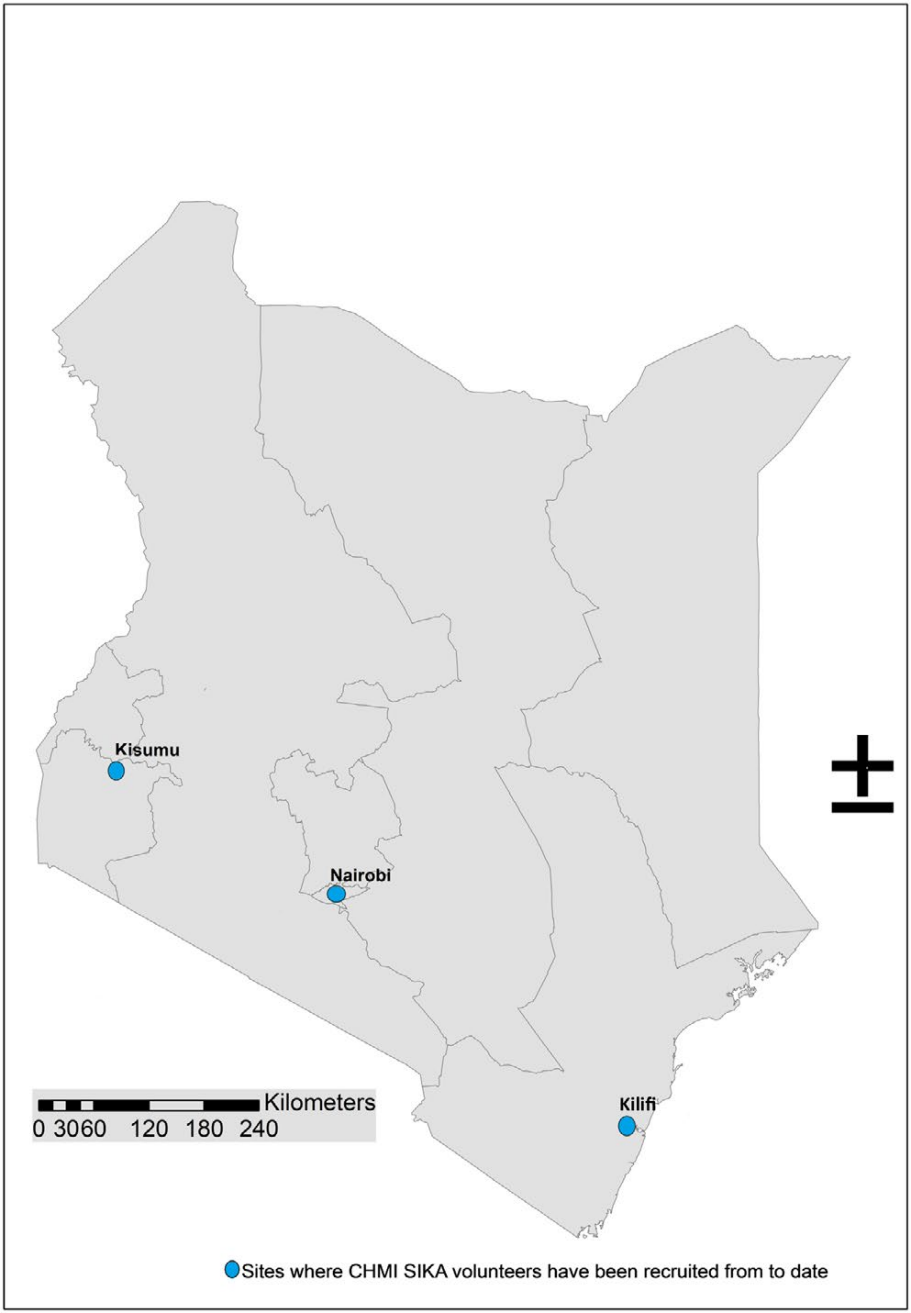
The three sites where CHMI SIKA volunteers have been recruited from to date [Colour figure can be viewed at wileyonlinelibrary.com]

KWTRP have conducted a series of CHMI studies (CHMI SIKA, or controlled human malaria infection in semi‐immune Kenyan adults) since 2013, as open label, non‐randomised trials involving semi‐immune Kenyan adults, and aimed at investigating how *in vivo* parasite growth rates of *Plasmodium falciparum* (Pf) are modified by pre‐existing immunity, measured by antibody levels to blood‐stage antigens.^23^ This programme of research has involved three phases, with Phase 1 conducted in Nairobi and Phases 2 and 3 at the KWTRP main hub in Kilifi. Each phase included a set of CHMI volunteers that were followed up to completion of their study participation before another phase was initiated. Phase 1 was a ‘proof of concept’ study in a setting where almost no malaria transmission occurs, and involved a small group of medical students.^24^ Phase 2 included volunteers from areas of low and moderate malaria transmission in Kilifi, and Phase 3 involved Kilifi residents as well as volunteers from a high malaria transmission area in Kisumu in Western Kenya.^25^ Figure 1 shows the geographic positioning of these sites. The first study on social and ethical aspects of CHMI SIKA was conducted during Phase 2.^26^ The qualitative study reported on in this paper was conducted within Phase 3, at which point, a total of 161 healthy volunteers had been recruited across the programme, aged between 18 and 45 years and with prior exposure and varying levels of immunity to malaria.

During CHMI SIKA Phase 3, volunteers found healthy on screening and who demonstrated understanding of the study were enrolled.^27^ An intravenous injection of *Plasmodium falciparum* sporozoites was administered on the day of enrolment into the study and volunteers admitted into an adapted residential facility at a nearby university. A 3‐day course of malaria treatment was given to those for whom (i) the density of infection exceeded a threshold (500 parasites per µl^28^) with or without symptoms, (ii) had symptoms and a blood film examination showed evidence of malaria infection, or (iii) no symptoms had developed by the study end‐point at 21 days. Overall volunteers were in residency at the university for between 15 and 24 days. The volunteers were reviewed at the research centre 35 days post‐infection.

### 2.2 The embedded empirical ethics study

The embedded empirical ethics study drew on a range of qualitative research methods, including in‐depth interviews, focus group discussions, non‐participant observations^29^ and study document review. For clarity, we refer to individuals joining the qualitative study as ‘study participants’ while those in CHMI SIKA are described as ‘volunteers’.

#### 2.2.1 Participants in the empirical ethics study

The empirical ethics study included a total of 69 participants, including CHMI SIKA volunteers (n=32), study staff (principal investigator, study clinicians and fieldworkers^30^) (n=17) and community representatives^31^ (n=20). Table 1 gives participants’ sociodemographic characteristics. In summary, participants who were CHMI volunteers were aged between 19 and 45 years, community representatives’ ages were from 27 to 73 years and the study staff from 30 to 48 years. More than half (53.3%) of CHMI volunteers participated in the empirical ethics study, consisting of all 15 CHMI volunteers from Kisumu and 17 of the 45 volunteers from Kilifi. The Kilifi volunteers who were not involved in the qualitative study were those who were symptomatic for malaria, on malaria treatment (and therefore preparing to exit the study) or declined to participate.^32^

**TABLE 1:** Characteristics of participants in the empirical ethics study embedded in CHMI SIKA Phase 3

Characteristics	Kisumu participants (n=15, 21.7%)		Kilifi participants (n=17, 24.6%)		Community Reps (n=20, 29%)		Study staff (n=17, 24.6%)			
	Female (n=7)	Male (n=8)	Female (n=9)	Male (n=8)	Female (n=8)	Male (n=12)	Female (n=4)	Male (n=10)	Female (n=2)	Male (n=1)
	(n, %)	(n, %)	(n, %)	(n, %)	(n, %)	(n, %)	(n, %)	(n, %)	(n, %)	(n, %)
*Age range*										
19–29	3 (43%)	4 (50%)	3 (33.3%)	3 (37.5%)	2 (25%)	0	0	0	0	0
30–40	3 (43%)	3 (37.5%)	3 (33.3%)	5 (62.5%)	1 (12.5%)	3 (25%)	3 (75%)	7 (70%)	1 (50%)	1 (100%)
41–51	1 (14%)	1 (12.5%)	3 (33.3%)	0	4 (50%)	2 (16.7%)	1 25(%)	3 (30%)	1 (50%)	0
52–62	0	0	0	0	0	5 (41.6%)	0	0	0	0
63–73	0	0	0	0	1 (12.5%)	2 (16.7%)	0	0	0	0
*Education level*										
None	0	0	2 (22.2%)	0	0	0	0	0	0	0
Primary education	1 (14.3%)	1 (12.5%)	5 (55.6%)	5 (62.5%)	6 (75%)	5 (41.7%)	0	0	0	0
Secondary education	4 (57.1%)	4 (50%)	1 (11.1%)	3 (37.5%)	2 (25%)	4 (33.3%)	4 (100%)	7 (70%)	0	0
Tertiary education	2 (28.6%)	3 (37.5%)	1 (11.1%)	0	0 (%)	3 (25%)	0	3 (30%)	2 (100%)	1 (100%)
*Occupation*										
None/volunteers	1 (12.5%)	0	4 (44.4%)	3 (37.5%)	0	1 (8.3%)	Study staff at the programme	Study staff at the programme	Study staff at the programme	Study staff at the programme
Students	0	3 (37.5%)	0	0	0	0				
Subsistence farmers	0	0	4 (44.4%)	0	4 (50%)	11 (91.7%)				
Self‐employed/business	5 (62.5%)	5 (62.5%)	0	4 (50%)	3 (37.5%)	0				
Employed	1 (12.5%)	0	1 (11.1%)	1 (12.5%)	1 (12.5%)	0				

KEMRI community representatives were selected purposively on the basis of residing in the geographic locations from which CHMI volunteers had been recruited. All CHMI study staff were invited to participate in the empirical ethics study. Five CHMI volunteers were followed up at home 8–9 weeks after exiting the study; this group were purposively selected on the basis of diversity in their social and research experiences to include: a female household head, a female participant who had experienced severe malaria symptoms and three individuals (one female and two male) whose families had not been supportive of their participation in the CHMI study.

### 2.3 Data collection

Data were collected mainly by IJ through observations, in‐depth interviews (eight) and focus group discussions (10), as summarised in Table 2. The interview guide^33^ was modified from a tool used during CHMI SIKA Phase 2,^34^ adapted for use with different participants.^35^ The tool was piloted before use and, as is typical for qualitative data collection, some of the questions were revised during data collection, informed by issues identified at debrief meetings following each interview. Most revisions were minor, for example adding prompts to clarify questions.

**TABLE 2:** Summary of data collection methods

Data collection method	Type of participants	Male	Female	Total
Observations	Throughout the conduct of study			
In‐depth interviews	study staff	1	2	3
Focus group discussions (4)	CHMI Volunteers	16	13	29
Follow‐up in‐depth interviews (5)	CHMI Volunteers	2^a^	3	3
Focus group discussions (3)	Field workers	11	3	14
Focus group discussions (3)	KCRs	11	9	20
Total		39	30	69

^a^ Although there were five in‐depth interviews held with CHMI volunteers, two of the male volunteers had previously been involved in the focus group discussions. They have not been counted as additional participants; thus only three new participants are counted for the in‐depth interviews.

Non‐participant observations (in which the researcher does not take an active role in the situation under scrutiny but carefully observes events, activities, and interactions in a non‐intrusive way)^36^ were undertaken throughout the study by IJ and DK, using a checklist to note issues around key areas of interest to the study (supplementary file 3). We held focus group discussions with CHMI volunteers from day 15 post‐infection. Five in‐depth interviews were held with CHMI study volunteers at their home localities two months after the study end, including two men who had earlier participated in a focus group discussion. Fieldworkers were involved in focus group discussions during the time of participant enrolment, as they could be reached easily at the same time and place. Focus group discussions with community representatives were held towards the end of the residency period.^37^

**FIGURE 2:**
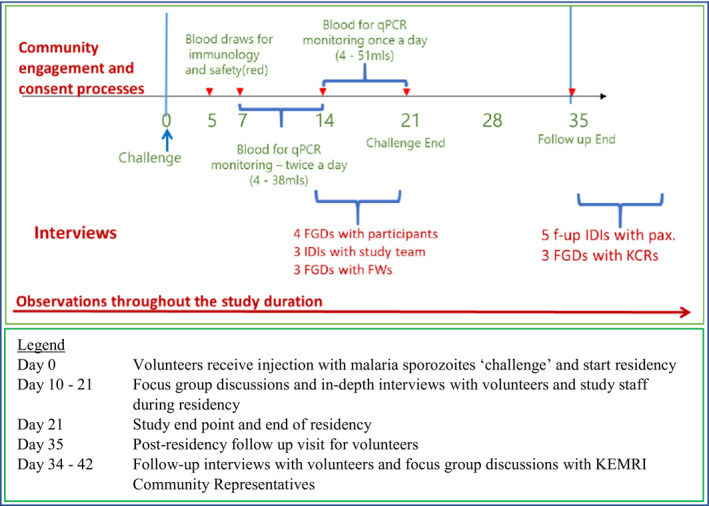
Data collection timelines for the empirical ethics study [Colour figure can be viewed at wileyonlinelibrary.com]

### 2.4 Data analysis

All interviews and group discussions were audio‐recorded. The data were transcribed verbatim, translated into English and managed with QSR Nvivo 10 software. Field notes were used to provide insights into the context and study‐related interactions. The data analysis followed a framework analysis^38^ approach, including the following steps: IJ, DK and PC reviewed and coded one transcript individually and later discussed it together, where they resolved any discrepancies in the coding and agreed on the initial coding framework. Three other transcripts were then coded by IJ and reviewed by this group to further refine the coding framework.^39^ All other transcripts were then coded by IJ and discussed with DK, and categories, themes and sub‐themes developed iteratively from codes, both inductively (from data) and deductively (drawing on ethics frameworks).^40^ Analysis charts were then drawn for comparison across themes and participant groups.

Throughout the tool development, data collection and analysis the researchers were aware of and tried to take account of their positionality, especially with regard to their roles as KEMRI staff, and their personal views on CHMI studies. For example, as KEMRI staff, the social value as well as potential sensitivities for the CHMI research approach were well recognised. For this reason, we aimed to be as reflexive as possible during data collection and analysis about our own potential influences, paying attention to the need to maintain a neutral stance, including probes around counter‐points during questioning, ensuring supportive dynamics within group discussion and working as a team to check emerging ideas and consider sources of influence. Additionally, IJ and DK had been introduced to the CHMI volunteers during screening, enrolment and study engagement activities, giving them an opportunity to build rapport prior to data collection.

### 2.5 Ethical considerations

The empirical ethics study was approved by the Kenya Medical Research Institute (KEMRI) Scientific and Ethics Review Unit (KEMRI/SERU/CGMRC/029/3190) and the Oxford Tropical Research Ethics Committee (OxTREC 2‐16). Information about the empirical ethics study was given to all volunteers at the time of screening for Phase 3 of the CHMI SIKA study and re‐emphasised during observation activities. Individual consent was sought at the time of interviews.

## 3 FINDINGS

Across the findings section, we first describe views around understanding and acceptability of CHMI SIKA, from the perspectives of different stakeholders in this study. Within this account, for participants who were CHMI volunteers, we note important changes in perceptions and attitudes over time, with a peak of anxiety at the time of sporozoites injection and later concerns about physical health related to symptoms developed as a consequence of the injection. We then present findings that highlight broad themes around the reasons that participants chose to volunteer for the CHMI study, given the unfamiliarity and concerns experienced, and their attitudes to joining a study like this again in future.

### 3.1 Understanding and acceptability of deliberate infection in CHI studies

#### 3.1.1 CHMI volunteers: learning about and joining the study

From our observations during the enrolment of volunteers into the CHMI main study and data gathered from interviews, most volunteers were excited about joining the study and described their involvement positively. This was partly attributed to being given detailed individual information and the comprehensive community engagement undertaken prior to and after enrolment, which had included visits to their homes by KWTRP fieldworkers who were also residents in their home areas. As in CHMI Phase 2, volunteers universally understood they would be infected with malaria parasites, might get sick as a result^41^ and would be treated under clinical observation until they recovered. They also knew, and valued, that they had been screened and found to be healthy prior to enrolment.

At the same time, most CHMI volunteers found the concept of research involving deliberate infection with a pathogen highly unusual, and this seemed to be linked to puzzlement and concerns about this approach to research, in spite of the information they had been given.

Okay, to me it’s quite eeh [long pause] … it doesn’t make sense … we’ve always known that … you are supposed to be given injection…to cure the disease that is already existing in your body. And now that I am just going to be given injection, to give me the disease? Yes? [laughing] So I had a different perception but all in all, I’m here, I’m participating and I’m a volunteer and I wasn’t forced. (F_34_Tertiary education_Employed)

In practice, unfamiliarity with the CHI study approach—and deliberate infection as a research strategy—was in keeping with the actual novelty of the approach in this setting. No studies in Kilifi over the last three decades have involved deliberate infection, prior to the initiation of Phase 2 CHMI SIKA. The numbers of volunteers involved in CHMI studies are also relatively small, and have included residents in specific geographic areas where previous KWTRP studies have characterised patterns of malaria endemicity.^42^ While careful stakeholder consultation activities had been undertaken ahead of planning for and initiation of CHMI SIKA, including KEMRI community representatives from the areas to be involved in the study, a wider awareness or more in‐depth consideration of this novel research approach within the community over time would, therefore, not have happened to any great extent.

#### 3.1.2 CHMI volunteers: being given an injection of malaria parasites

While, as above, participants in our study were initially excited about joining CHMI SIKA, most described heightened concerns about risks of deliberate infection immediately before and during the actual injection of malaria sporozoites:

… Now for me during that time they were inserting the parasites, first when I was giving them my hand to be injected, I was sweating … I did not take it lightly, I took it as something very serious, I’m fine [due to screening], I’ve been examined, but right now I am being infected with a disease … (M_25_Secondary education_Unemployed)

A specific new concern emerging at the point of injection with sporozoites concerned the amount of malaria to be injected, related to perceptions of safety. Some linked the size of the malaria ‘dose’ to that of the syringe used, and contrasted this to an imagined size of a malaria parasite or the amount of malaria that a ‘tiny mosquito’ might carry. As well as worries about the malaria dose being too high for safety, perceptions of a mismatch between the size of a mosquito and the syringe led to doubts that the injection might not contain malaria sporozoites, but something more harmful:

… the mosquito is as small as this and the drug [the inoculant] is a lot more than the mosquito [Clicks tongue] ‘Is this really malaria?’ I questioned myself within my heart ‘Is this really malaria?’ Then I just said it’s okay, let them inject me. Yes, I have brought myself… It disturbed me and up to now it is still disturbing me … (M_21_Tertiary education_Student)

Countering these immediate worries for most volunteers was the presence and perceived diligence of study clinicians and monitoring staff, where the latter observed injection preparation closely, handed syringes directly to study clinicians and were at hand to respond to issues. A further quality check in place was that clinicians sought confirmation from each volunteer that they were willing to continue with participation before giving the injection with sporozoites. This additional checking was reassuring to most volunteers, but for others increased their anxieties, leading them to worry that high levels of monitoring and checking implied higher levels of risk than had been envisaged:

For me initially I was scared because when she [research clinician] said ‘If you don’t want to be injected with the parasites you’d better say that you don’t want’. She repeated those words three times, so I said eeh, and the way she is just repeating … [I thought] won’t this thing just go and harm us completely? As in our bodies will be at risk of harm, maybe it can even make me lose my life. (M_24_Secondary education_Businessman)

Anxieties were further heightened by the interpretation of a number of observations made around the time of injection. Firstly, the inoculant was prepared in a different room to that where the injection was given, so not visible to the volunteer. Secondly, there was a perceived inconsistency in the direct monitoring of the preparation and injection of the inoculant, so that monitoring was reportedly in place for some volunteers but not others. Thirdly, the movement in and out of rooms seems to have been disturbing, and was associated with fears that decisions were being taken about the dose of sporozoites to be given to different volunteers, including that some might be given a higher dose than others:

… Because I saw like it was Xxx [research staff] who was bringing them [syringes with inoculant] one by one … from the room where that Mzungu [white man] was … You know there is no day that I had seen her going inside … so the way I took it is that she was aware of the limits, that she knows maybe [another volunteer] is not to get as many [parasites] as mine, you see? (M_21_Tertiary education_Farmer)

Across the groups, the immediate concerns about being injected with malaria were much more common amongst volunteers from Kisumu in Western Kenya, who also had less experience and connection with KWTRP and its staff than those resident in Kilifi. Amongst volunteers from Kilifi, those who had participated in KWTRP research projects in the past, or had family members who had done so, showed less anxiety around the study than their fellow community members who had not previously participated in research. Illustrating the same point around the role of familiarity, some participants wondered whether studies could use a research approach based on more familiar techniques, for example, using mosquito bites^43^ as a mechanism for deliberate infection, rather than injection with sporozoites.

#### 3.1.3 CHMI volunteers: Concerns during the post‐infection period

Throughout the study, a minority of volunteers developed symptoms of malaria and were treated following the study protocol. Issues of trust again emerged at this point, where those who did not develop symptoms began to wonder whether they had really been injected with malaria parasites:

… am thinking that having already been injected they told us we shall get dizzy, and when I ask them they tell me not yet. So, I am wondering was it really malaria or…? (_F_37_secondary education_Businesswoman)

For those who did develop symptoms, some experienced greater levels of discomfort than had been expected:^44^

Weee! When I got the malaria that is when I realised malaria is not a joke! … so it was true that we were to get malaria! We did not know we would truly get malaria. We did not expect this malaria of being injected would be severe, you see. Yes… I felt like my head was falling [laughing]. (F_29_Tertiary education_Volunteer)

The severity of the symptoms experienced led one volunteer to feel she would not participate in future similar studies:

Considering my blood, I don’t think I can participate again [giggles] … It seems my blood doesn’t want any foreign thing added to it …I mean, I have been thinking like that because there were many [volunteers] who remained okay but as for me I got problems… but as for other studies I can participate. Yes. (F_34_No education_Casual employment)

#### 3.1.4 CHMI research staff: conflicting views

Amongst the research team for CHMI SKIA, clinical staff expressed views that highlighted both a positive attitude towards the approach and some underlying discomfort. In this way, most research staff felt that the careful study design and conduct in a malaria endemic setting limited risks and justified conducting this research in Kenya, particularly given its high social value in this particular population:

I feel to do these studies in an endemic population is actually far much safer than if you are doing it on somebody who is completely naïve. Because the goodness with an endemic population is that they already have pre‐exposure which might actually cushion them from severe disease. (F_38_Tertiary education_Study staff)

At the same time, clinical research staff appeared uncomfortable in responding to specific questions on the acceptability of deliberate infection; some felt that deliberately infecting healthy volunteers with a pathogen was in tension with their clinical responsibilities to cure and ‘not harm’. According to one research clinician, the extent of research, as opposed to clinical experience, would influence the way clinical staff might react to a proposal of deliberate infection. At the same time, the framing of this response suggests difficulty in ‘accepting’ this role:

I'm not the right person to be asked that question because you are asking someone who has been in research for almost 12 to 13 years, because I’m prepared at this level academically, professionally to accept it. If I would have been [a staff member] coming from a purely clinical aspect I would have been … a bit taken aback … (M_39_Tertiary education_Study staff)

Another clinical researcher with less than five years’ experience in research felt uncomfortable in responding to the question and instead changed the question to discuss altruism as a good reason for participation:

For me as a person I think it’s not bad [to be deliberately infected] because you are, you are, you are contributing to the overall, overall benefits for our own good, our public good. (F_35_Tertiary education_Study staff)

CHMI study fieldworkers were more forthcoming with their opinions about the acceptability of deliberate infection. Their study roles included introducing CHMI SIKA 3 to community members in the geographic areas targeted for recruitment (known low, moderate or high transmission levels) and following volunteers up in their homes after exiting the study. Three of the fieldworkers, as local residents themselves, had been volunteers in CHMI SIKA 2. The views raised within this group reflect both a level of discomfort with and support for the study approach, based on different types of arguments, highlighting potential tensions for this group of study staff. Some concerns centred on fairness, linked to the idea of ‘using’ people in ways that do not take account of their human nature as well in the distribution of benefits:

… in my own opinion being injected with malaria parasites … personally I felt that it’s not humane because you’re using the body of another human to get, let us say, like for example, business because maybe after you studied the research and got the research findings … you will have benefited yourself but the one you have used in the study did not benefit from anything like that … (M_36_Secondary education_Study staff)

A different kind of attitude expressed was one of strong positive support for the study (CHMI SIKA) as a ‘professional responsibility’ to the research institution (‘spreading the gospel’):

Okay for me; not as an officer [staff] but as a person, because if I talk as an officer, I will automatically support KEMRI. So let me talk like P2 [Identification used for the interview]. According to my understanding, I support this challenge … I continue spreading this message, the gospel of CHMI. (F_32_Secondary education_Study staff)

The context for this particular CHMI study was critical to the support that study staff felt able to give. Staff were specifically reassured by the knowledge that deliberate infection was of a short‐lived infection (malaria) which could be quickly treated with safe and effective treatment, and that high‐quality care was provided for all volunteers throughout the study. Of the staff groups, fieldworkers seemed to feel most conflicted about the research approach. While they had good knowledge of the study protocol and potential social value, and were routinely involved in information giving to prospective volunteers, more than half remained cautious about the risks of this type of study. These voices of caution included some fieldworkers who had themselves been volunteers in CHMI SIKA 2.

That fear is usually there because like me I used to think that, okay, we were told that it is like 99% safe. But there is this 1% that we are not sure of. What if this 1% falls on me? That fear is there. It is not for me alone, but also other people usually have that fear. (M_32_Tertiary education_Study staff)

For similar reasons, other study staff (including fieldworkers) indicated that they would routinely go out of their way to attend to volunteers’ needs beyond study requirements, as a means of ensuring their safety and wellbeing. Similarly, study staff who were in constant contact with volunteers as part of ensuring continuous medical supervision took on much wider roles as counsellors, peacemakers and problem solvers addressing issues such as boredom, worry about family issues that could not be attended to and sadness associated with ‘missing’ being with loved ones, including family and friends, over the period of residency.

#### 3.1.5 KEMRI community representatives

Community representatives had heard about the CHMI study during routine quarterly meetings with KWTRP community liaison staff held as part of KWTRP’s strategic community engagement programme as well as in study‐specific community engagement forums. While some could remember some elements of the CHMI study, for most participants, deliberate infection was clearly a new concept when discussed during the focus group discussions. As with other participants, views on the acceptability and safety of deliberately infecting healthy volunteers with malaria were mixed, both across the group and for individuals. In this way, while some raised concerns around the concept of deliberate infection, more than half of them felt that the CHMI research approach could be important locally in terms of tackling an important cause of disease and deaths. This group emphasised the importance of providing adequate information about the study to community representatives and to individual potential volunteers. In doing so, there seemed to be gaps in understanding of a right to withdraw from research, as the first of the following quotes suggests:

Even me I don’t want [to be enrolled into a CHMI study], and for me to accept it’s very difficult, but if you volunteer saying, ‘Let whatever happens be’, because if you go there you have agreed for the research to be conducted on you, you must accept everything. You can be given malaria injection and it can be very serious in your body and you will be told that ‘You accepted yourself because you even signed here. Yes, you signed yourself here’. (M_62_Tertiary education_Farmer)

It’s … it is not that easy with our bodies but because the research has to be done for us to get better drugs to eradicate specifically or to treat malaria so that it is not a threat to people the way it is now; it is okay for research to be done. But someone will think it’s hard; because just thinking about it, you’ll feel it is difficult, but if you sit down and reflect on it further it is very okay. (F_63_Primary education_Farmer)

### 3.2 Why then participate in CHMI SIKA?

Given the concerns and worries raised by volunteers about the idea of being given a deliberate malaria infection, we sought to find out why people had joined the study and stayed until the end. Overall, the findings from this and our earlier study^45^ show that, in deciding whether to participate, volunteers worked through a process of weighing up the benefits of participation to them, their families and the wider community (including the wider social value in contributing to vaccine development), as well as the concerns we have described so far about being given a ‘deliberate infection’ of malaria and a number of physical, emotional and social burdens or harms that were anticipated at the point of joining. In summary, the underlying reasons for participating fell into four main areas; (i) altruistic reasons, (ii) compensation as described in our previous publication, (iii) trust in the research institution and in researchers, and (iv) trust in the protocol review processes.

Almost all respondents across all groups expressed strong altruistic motivations for participation, but more so the Kisumu participants who came from a highly malaria endemic area, and felt that the outcomes of the study would be of benefit in the future both locally and internationally.

I have a passion for children and pregnant women. I wouldn’t like to see pregnant mothers die and children under five who are vulnerable to malaria die just because we lack a proper vaccine to prevent malaria. And that is why I was driven to participate so that we could find a new way, I mean, a vaccine or a drug that could reduce the mortality rate, yes … That is the thing that gave me the motivation that no matter the kind of project I can enrol because I want them to get the treatment or prevention of different types of illness found locally and commonly. (M_42_Secondary education_Businessman)

Compensation as earlier stated also emerged as a strong motivating factor for joining CHMI SIKA for both male and female volunteers of all ages, including those who held strong altruistic views, as indicated in the following quote:

I’ll still revolve around my issue of the power of the dollar… it will always carry the day because I believe if at all there was no value attached to our being here then nobody would have been injected with malaria and then there is nothing coming out of it. (M_39_Secondary education_Farmer)

Most had planned for the money; viewing compensation as a means of meeting immediate pressing family needs they would not otherwise have managed to accomplish had it not been for their participation in the study.

… I have a wife and my children and am still in my father’s home. So I have worked hard to get some other money so that I can move out and build my own home, but my financial situation has been very difficult. So when I heard about this [CHMI] I had to go and sit with my wife; we talked and I explained that we may be away for one month and if it will be our luck we will get that money and build our home. (M_38_Primary education_Tailor)

Also as alluded to in earlier sections, trust in the research institution was a key influence on decision‐making, given concerns about safety and the need for reassurance that KEMRI could be trusted to guarantee this. Perceptions of trust towards KWTRP was described based on its positive reputation for research and supporting healthcare, its long‐standing presence and positive relations in the community, and a belief that it would not intentionally harm people in the community. Interestingly, some volunteers had learnt about the CHMI study from relatives who had participated in earlier phases, and were reassured by this knowledge:

I too did not have any doubts because in the second group my nephew was here, my brother was also here and they came and participated and went back home, the parasites were not detected and they got the dose [malaria treatment] and up to now they have not been sick and do not have any problem. (F_42_Primary education_Farmer)

About a half of the respondents across all the groups, including those who had never participated in research before, also spontaneously reported *trusting the research review processes* in the country. Information about the review process was included in the consent forms, was discussed during information giving and community engagement processes and a telephone number to the national research regulation office included in the consent form.

#### 3.2.1 Future CHI: participating and supporting

It is clear from our data that the reality of some of the burdens (for example, the levels of anxiety associated with being given an infection, the discomfort of having frequent blood sampling and the discomfort and anxiety associated with having an acute malaria infection) had not always been fully taken into account during decision‐making. In future publications we aim to explore these issues in more depth, particularly around decision‐making on participation in relation to risks of undue inducement, and forms and levels of physical, social and emotional burdens or harms experienced by volunteers, including longer term follow‐up data.

Arguably as a ‘marker’ for ways in which volunteers weighed up their experiences of CHMI SIKA overall, many expressed interest in participating in future similar studies, that is, research involving deliberate infection with an acute and treatable pathogen:

I think I would participate again in another exercise. At first it was a risk taking, but now I feel I can withstand the temperature. So, I am prepared, I know the corners so I am not moved … . (M_39_Secondary education_Farmer)

CHMI SIKA volunteers and other participants in our study also commented on the importance of wide community engagement in underpinning the acceptability of research based on ‘deliberate infection’ given the unfamiliarity of this approach. Key messages for engagement would need to be developed around the justification for the approach and volunteers’ safety and levels of risks of the study to avoid generating new or heightening existing rumours and misconceptions about research. Fieldworkers, as members of the community themselves, were particularly worried about damage to their reputation should the CHMI study cause problems in the community. Highlighting that CHMI studies were being importantly justified through their putative social value, participants strongly recommended that any vaccine candidates discovered through this route should be quickly moved into trials to accelerate the vaccine development process.

## 4 DISCUSSION

CHI studies in LMICs are relatively new, have the potential to accelerate discovery of much needed therapies including vaccines, and present a range of ethical issues, as outlined in the introduction to this paper. The potential social value of the research has been argued as a core justification for conducting CHI studies in LMIC for diseases endemic in these settings.^46^ Other arguments are that CHI studies are cost‐effective as they involve significantly smaller numbers of volunteers over shorter durations compared to Phase III clinical trials;^47^ and can inform early whether a therapy could potentially be efficacious and thus can sieve out those potential therapies that are less/not likely to lead to therapies early on.^48^ Taken together, these arguments provide a strong rationale for conducting CHI studies in settings where conditions are endemic, as is the case for malaria in our context.

In this paper, we draw on our experiences of conducting CHMI studies in coastal Kenya to contribute to the debate on the acceptability of ‘deliberate infection’ as an essential component of CHI studies. In particular, we draw on our findings on research stakeholders’ perceptions and experiences of deliberate infection in a specific CHMI study conducted at a long established international collaborative research programme in Kenya. As outlined in the introduction to this paper, an important argument for acceptability is that the process of deliberate infection ensures that a well‐considered amount of pathogen is carefully administered to the volunteers using a carefully considered route of administration and conducted under high level of diligence.^49^ Within this argument, volunteers’ safety and wellbeing is paramount in determining acceptability of deliberate infection in these types of studies.

In this discussion we aim to underline findings that showed that the fact of being deliberately infected (by injection in this case) with a disease‐causing organism can have an impact on volunteers that may go beyond biomedical understandings of safety but are nonetheless critical aspects of wellbeing, and have a moral importance for arguments about the acceptability of deliberate infection. In our study, deliberate infection was seen as a highly unusual research strategy amongst volunteers and the community representatives. As we have shown, and go on to discuss, the notion engendered sometimes very high levels of anxiety over the course of participation, alongside experiences of ill‐health—where malaria infection followed—which were at times perceived as very severe. We note that the individual psychological experiences of excitement, anxiety and fear fluctuated strongly over the period of participation, primarily in relation to specific research activities, but also influenced by social relations between volunteers and staff. Similarly, over prolonged periods of residency, psychological and emotional burdens were common, and responded to by the study team.

### 4.1 Tensions and fluctuations within emotional responses to being a volunteer in the CHMI study

In this and our earlier paper,^50^ we have described that volunteers’ strong positive interest in and, for some, excitement about participation in CHMI SIKA was often continuously counterbalanced by fears and anxieties about participating in an unfamiliar form of research, with uncertain outcomes and the possibility of significant illness resulting. The findings show the way that physical, psychological and emotional burdens of participation fluctuated strongly over time, but for some people and at certain times were particularly severe. For example, excitement was high at the point of recruitment, potentially accentuated by the fact that only a minority of people who had shown interest in participation at the outset were eventually recruited following screening processes.

The most intense anxiety and fear for many volunteers was associated with the act of being injected with malaria parasites. At this point, trust was enacted positively for some, who felt reassured that KWTRP staff would not deliberately harm them, and was clearly a more ambivalent feature for others, who, for example, worried about the nature and the amount of the inoculum. Staff practices introduced to support procedural quality, such as the double checking of syringes and checking volunteers’ willingness to continue with participation, acted both to reassure some volunteers and worry others, highlighting the challenges of anticipating and responding to volunteers’ concerns over time. Similarly, the disappearance of staff from the room when preparing injections acted as an unexpectedly negative influence on confidence and trust.

### 4.2 Physical, emotional and social harms

Volunteers in CHMI SIKA who participated in our study described a range of physical, emotional and social burdens or harms associated with the period of residency required in this study. We particularly note the fluctuating nature of these harms over time and the dissonance between a theoretical understanding of the procedures involved in the study and the experienced level of burdens in relation to certain elements. Particular examples are the sometimes very high levels of anxiety around being ‘deliberately injected’ with malaria, the unanticipated level of suffering associated with an acute malaria infection. Against this background, study staff who were in constant contact with volunteers as part of ensuring continuous medical supervision took on much wider roles as counsellors, peacemakers and problem solvers. Their presence during the residency was critical to the wellbeing of volunteers as well as the smooth continuation of the CHMI study. Similarly, fieldworkers described their roles in reassuring volunteers, and making follow‐up visits after residency to satisfy themselves that their fellow community members were unharmed. It could be argued that the roles of staff in practice far exceeded what was anticipated, and that it was essential that these individuals had the capacity to respond effectively to demands outside their professional competencies, and over quite long periods of time (as, in fact, they did).

Across these findings, it seems that the arguments for the acceptability of deliberate infection in the literature on the basis of the existence of high standards of safety around the research procedures involved may not go far enough in responding to the actual experiences of volunteers in CHMI studies. In addition to safety, we propose that acceptability should be contingent on recognising and responding to a range of serious burdens experienced by volunteers that are directly related to ‘deliberate infection’ and that go beyond physical safety but are central to wellbeing in important ways. Drawing on our findings, we conclude with some recommendations for CHMI studies that we believe will help to address the risks of additional and potentially unnoticed physical, psychological and emotional burdens that research based on ‘deliberate infection’ may entail:

1. Strengthening informed consent processes, including seeking ways of building a more realistic understanding of the implications of participation, particularly in relation to the procedures such as deliberate infection, repeated blood sampling, the physical implications of developing an acute malaria infection and the social and emotional costs of prolonged residency. Specifically, we suggest that ICFs should include a statement on these psychological issues or social harms volunteers may experience at different time points while participating in a CHMI study.
2. Ensuring that responsive forms of communication and emotional support are available to volunteers throughout the study as much as possible, but particularly during periods when extreme anxiety or other forms of distress are likely. Importantly, since many of these types of burdens are likely to fluctuate over the period of participation, the processes put in place to address these challenges must also be responsive over time. Research and monitoring activities in a different setting are likely to be important to build context‐specific and locally responsive policies and procedures to minimise harms and burdens for participants, including additional counselling and practical/environmental support.
3. It is possible that deliberate infection, while otherwise ethically permissible, can be morally problematic for some physician‐researchers, as it contravenes their clinical training and their duty of care to alleviate, rather than cause, suffering and harm.^51^ Our attempt at investigating this issue was not conclusive in a regulatory or normative sense, but revealed a number of issues worth considering in future studies, including that long‐term physician‐researchers who are primarily researchers may have different views about deliberately infecting patients compared to those with limited or no research experience; and that physician‐researchers may experience a moral dilemma, but not feel able to express their concerns. A practical suggestion is that clinical research staff and fieldworkers should not only be supported to develop skills to recognise and respond to volunteers’ emotional burdens, but should themselves be supported in resolving the tensions they may experience between research and clinical or community roles and responsibilities associated with CHMI.

## 5 CONCLUSION

The concept of deliberate infection core to CHMI is uncommon in practice and unfamiliar to the lay public in LMICs. The literature sources acknowledge that deliberate infection carries a risk that unpleasant symptoms and anxieties might be experienced by volunteers, and that the carefully controlled conduct of CHI minimises the extent to which harms and discomforts might be experienced. We describe findings from an empirical ethics study on research stakeholders’ perceptions and experiences of deliberate infection in CHMI study, including CHMI volunteers, study staff and community representatives.

Decisions to join and remain in the study hinged on familiarity with malaria as a common and treatable condition, levels of compensation, altruism, observation of the experiences of earlier volunteers and trust in the expertise of the study team, the research programme, and the research review system. Volunteers’ excitement of being involved in the study, and their hopes and fears in relation to this, shifted in nature, extent and focus over the course of the study, reflecting the unfamiliarity of some research processes involved. Amongst those CHMI volunteers who developed malaria symptoms, some experienced severe physical discomfort; volunteers’ burdens also included fluctuating but sometimes severe forms of psychological and emotional reactions across the duration of residency. Heightened levels of anxiety occurred particularly during the injection with malaria parasites, suggesting high levels of discomfort with this CHMI study procedure. These findings highlight the importance of understanding grounded realities for volunteers over time in defining the ethical dimensions of CHI.

We argue that researchers planning for CHMI studies and reviewers should consider both the physical, social and psychological burdens of participation when assessing levels of risk for these studies. We further argue that ethics oversight of CHMI studies should take account of these issues, in deciding whether consent, engagement and the balance of benefits and harms for a given CHMI are reasonable in a given context. To address these issues, we suggest that skilled counselling services should be provided both for CHMI volunteers, and for study staff who may experience tensions in their dual roles as clinicians and researchers. Capacity building in communication and management of emotions may be important for study staff given the extra responsibilities they take on to offer emotional support to volunteers throughout the study.

## Conflict of interest

The authors declare no conflict of interest.

## Supporting information

Appendix 1

Appendix 2

Appendix 3

Appendix 4

Appendix 5

## Footnotes

^1^ Sauerwein, R. W., Roestenberg, M., & Moorthy, V. S. (2011). Experimental human challenge infections can accelerate clinical malaria vaccine development. *Nature Reviews Immunology, 11*(1), 57.

^2^ Hodgson, S. H., Juma, E., Salim, A., Magiri, C., Kimani, D., Njenga, D., … Awuondo, K. (2014). Evaluating controlled human malaria infection in Kenyan adults with varying degrees of prior exposure to Plasmodium falciparum using sporozoites administered by intramuscular injection. *Frontiers in Microbiology, 5*, 686; Kapulu, M. C., Njuguna, P., Hamaluba, M., & Team, C.‐S. S. (2018). Controlled human malaria infection in semi‐immune Kenyan adults (CHMI‐SIKA): A study protocol to investigate in vivo Plasmodium falciparum malaria parasite growth in the context of pre‐existing immunity. *Wellcome Open Research, 3*; Kraft, S. A., Duenas, D. M., Kublin, J. G., Shipman, K. J., Murphy, S. C., & Shah, S. K. (2019). Exploring ethical concerns about human challenge studies: A qualitative study of controlled human malaria infection study participants’ motivations and attitudes. *Journal of Empirical Research on Human Research Ethics, 14*(1), 49–60.

^3^ Selgelid, M. J., & Jamrozik, E. (2018). Ethical challenges posed by human infection challenge studies in endemic settings. *Indian Journal of Medical Ethics, 3*(4), 263–266.

^4^ Hodgson, S. H. et al. *op. cit*. note 2; Collins, K. A., Wang, C. Y., Adams, M., Mitchell, H., Rampton, M., Elliott, S., … Chalon, S. (2018). A controlled human malaria infection model enabling evaluation of transmission‐blocking interventions. *Journal of Clinical Investigation, 128*(4), 1551–1562.

^5^ Selgelid & Jamrozik, *op. cit*. note 3; Spring, M., Polhemus, M., & Ockenhouse, C. (2014). Controlled human malaria infection. *Journal of infectious diseases, 209*(suppl_2), S40–S45; Miller, F. G., & Grady, C. (2001). The ethical challenge of infection‐inducing challenge experiments. *Clinical Infectious Diseases, 33*(7), 1028–1033.

^6^ Hope, T., & McMillan, J. (2004). Challenge studies of human volunteers: ethical issues. *Journal of Medical Ethics, 30*(1), 110–116.

^7^ Selgelid & Jamrozik, *op. cit*. note 3; Miller & Grady*, op. cit*. note 5.

^8^ Dholakia, S., & Dholakia, S. (2018). Conducting controlled human infection model studies in India is an ethical obligation. *Indian Journal of Medical Ethics, 111*(4), 279–285.

^9^ Selgelid & Jamrozik, *op. cit*. note 3; Miller & Grady, *op. cit*. note 5.

^10^ Selgelid & Jamrozik, *op. cit*. note 3.

^11^ Hodgson, S. H., Juma, E., Salim, A., Magiri, C., Njenga, D., Molyneux, S., … Marsh, K. (2015). Lessons learnt from the first controlled human malaria infection study conducted in Nairobi, Kenya. *Malar J, 14*, 182; Selgelid & Jamrozik, *op. cit*. note 3; Bambery, B., Selgelid, M., Weijer, C., Savulescu, J., & Pollard, A. J. (2015). Ethical criteria for human challenge studies in infectious diseases. *Public Health Ethics, 9*(1), 92–103.

^12^ See for example https://www.who.int/malaria/media/malaria‐vaccine‐implementation‐qa/en/

^13^ Jamrozik, E., Fuente‐Núñez, V., Reis, A., Ringwald, P., & Selgelid, M. J. (2015). Ethical aspects of malaria control and research. *Malaria Journal, 14*(1), 518.

^14^ Hodgson, S. H. et al. *op. cit*. note 2; Kapulu, M. C. et al. *op. cit*. note 2.

^15^ Hodgson, S. H. et al. *op. cit*. note 2.

^16^ Hope & McMillan, *op. cit. *note 6.

^17^ Miller & Grady, *op. cit*. note 5.

^18^ Hope & McMillan, *op. cit. *note 6.

^19^ Njue, M., Njuguna, P., Kapulu, M. C., Sanga, G., Bejon, P., Marsh, V., … & Kamuya, D. (2018). Ethical considerations in controlled human malaria infection studies in low resource settings: Experiences and perceptions of study participants in a malaria challenge study in Kenya. *Wellcome Open Research, 3*.

^20^ Musschenga, A. W. (2005). Empirical ethics, context‐sensitivity, and contextualism. *Journal of Medicine and Philosophy, 30*(5), 467–490; Wangmo, T., Hauri, S., Gennet, E., Anane‐Sarpong, E., Provoost, V., & Elger, B. S. (2018). An update on the ‘empirical turn’ in bioethics: Analysis of empirical research in nine bioethics journals. *BMC Medical Ethics, 19*(1), 6.

^21^ Kraft, S. A. et al. *op. cit*. note 2.

^22^ Scott, J. A. G., Bauni, E., Moisi, J. C., Ojal, J., Gatakaa, H., Nyundo, C., … Marsh, K. (2012). Profile: The Kilifi health and demographic surveillance system (KHDSS). *International Journal of Epidemiology, 41*(3), 650–657.

^23^ Kapulu, M. C. et al. *op. cit*. note 2.

^24^ Hodgson, S. H. et al. *op. cit*. note 2; Hodgson, S. H. et al. *op. cit*. note 11.

^25^ Kapulu, M. C. et al. *op. cit*. note 2.

^26^ Njue, M. et al. *op. cit*. note 19.

^27^ Ibid.; see supplementary file 2 for test of understanding tool.

^28^ A threshold substantially lower than 2500 parasites per µl at which clinical illness becomes more common in children in Kenya.

^29^ Non‐participant observation was done at screening, enrolment, during residency and on day 35 post‐infection review visit.

^30^ Fieldworkers are full time employees at the research programme who are secondary school leavers and resident within the communities where research is conducted.

^31^ KEMRI community representatives are a group of typical community members who have been selected by their own communities to interact regularly with the Community Liaison Group in the research programme to support the community engagement agenda.

^32^ The main reason for declining to join was an unmet expectation that their participation in the qualitative study during the period of residency would involve compensation for time.

^33^ See supplementary file 1 for the interview guide with CHMI volunteers.

^34^ See supplementary file 2 for the social science studies generic focus group discussion guide.

^35^ For example, the PIs guide had additional questions on the basis of the social science study, general experiences of leading the study and community engagement activities.

^36^ Spencer, S., Stone, T., Kable, A., & McMillan, M. (2019). Adolescents’ experiences of distress on an acute mental health inpatient unit: A qualitative study. *International Journal of Mental Health Nursing, 28*(3), 712–720; Theobald, K. A., & Ramsbotham, J. (2019). Inquiry‐based learning and clinical reasoning scaffolds: An action research project to support undergraduate students' learning to ‘think like a nurse’. *Nurse Education in Practice, 38*(July 2019), 59–65.

^37^ See Figure 2 for data collection timelines for this empirical ethics study.

^38^ Green, J., & Thorogood, N. (2018). Framework analysis. In *Qualitative methods for health research* (4^th^ ed,) (pp 268–272). London: Sage.

^39^ See appendix 4 for the coding framework.

^40^ Srivastava, A. and S. B. Thomson (2009). Framework analysis: A qualitative methodology for applied policy research. *Journal of Administration & Governance*, *4*(2), 72–79.

^41^ Njue, M. et al. *op. cit*. note 19.

^42^ Bejon, P., Williams, T. N., Liljander, A., Noor, A. M., Wambua, J., Ogada, E., … Färnert, A. (2010). Stable and unstable malaria hotspots in longitudinal cohort studies in Kenya. *PLoS medicine, 7*(7).

^43^ Sauerwein, R. et al. *op. cit*. note 1.

^44^ No volunteers developed symptoms that were classed as ‘severe adverse events’ according to study protocol.

^45^ Njue, M. et al. *op. cit*. note 19.

^46^ Selgelid & Jamrozik, *op. cit*. note 3; Rid, A. (2014). Setting risk thresholds in biomedical research: lessons from the debate about minimal risk. *Monash Bioethics Review, 32*(1–2), 63–85; Hope & McMillan, *op. cit*. note 6.

^47^ Dholakia and Dholakia, *op. cit*. note 8; Selgelid & Jamrozik, *op. cit*. note 3; Hodgson, S. H. et al. *op. cit*. note 2; Gordon, S. B., Rylance, J., Luck, A., Jambo, K., Ferreira, D. M., Manda‐Taylor, L., …, & Seager, Z. (2017). A framework for controlled human infection model (CHIM) studies in Malawi: Report of a wellcome trust workshop on CHIM in low income countries held in Blantyre, Malawi. *Wellcome Open Research, 2*.

^48^ Miller & Grady, *op. cit*.* *note 5.

^49^ Gordon, S. B. et al. *op. cit*. note 48; Selgelid & Jamrozik, *op. cit*. note 3; Spring, M. et al. *op. cit*. note 5.

^50^ Njue, M. et al. *op. cit*. note 19.

^51^ Hope & McMillan, *op. cit. *note 6; Miller & Grady, *op. cit. *note 5.

