# Supplementary material for: Deliberately infecting healthy volunteers with malaria parasites: Perceptions and experiences of participants and other stakeholders in a Kenyan‐based malaria infection study
Source: Bioethics. 2020 Jul 9;34(8):819–32. doi: 10.1111/bioe.12781 (PMC7689838; doi:10.1111/bioe.12781)
Supplement: Supplementary file 1 — Appendix 1 [file BIOE-34-819-s001.docx]

**APPENDIX 1**

**TOPIC GUIDE FOR DISCUSSIONS WITH CHMI PARTICIPANTS (FGDS)**

**A. FAMILIARIZATION WITH PARTICIPANTS/ICE BREAKER**

1. Who informed you about this malaria challenge study?
2. What information were you given about this study before starting the screening process?

***(Probe for* what is the study aiming to find out, *screening, consent processes, challenge processes, end of study)***

**B. INFORMED CONSENT PROCESS**

1. Thinking about all the information giving/screening/consenting sessions you have attended **a)** what are your views about the information given during the information giving sessions? **b)** What was done differently in those different visits? **c)** Was the information given by clinicians and FWs the same?

***(Probe about clarity of the information they were given, how consistent the messaging was, were they able to understand the information? e.g. explanations and demonstrations on blood draw).***

1. Please mention what you know about what the study involves.

***(Do not probe; participants to spontaneously mention screening, consenting, enrollment, in-patient confinement at Pwani Univ., injection with parasites, blood draws and monitoring, ending of study)***

1. What are some of the reasons one may not be able to participate in the study after going through screening? How do you feel about that?

***(Probe for screening failure or qualifying at screening, but not being called to participate).***

**C. DECISION MAKING**

**a) Motivating factors and barriers (20 min)**

1. What are the reasons that made you want to participate in the malaria challenge study?

(If not mentioned spontaneously probe on altruism, compensation)

1. How did the requirements for participating in the study affect your decision making? (***Probe about requirements for participation e.g. use of ITNs, for female participants use of contraceptives, staying 3 extra days when requiring to leave the study)***
2. How do you feel about being in the study? Any motivating factors? Do you have any regrets? Any change in perceptions between before joining the study and now? ***(Probe for likes, dislikes, concerns or fears, in comparison to those at home not in the study)***
3. What importance/value do you think participants place in their involvement in such a challenge study? How does that affect perceptions towards involvement in the study? How does it affect their motivation to participate in the study?

**b) Involvement of family members/significant others (30 min)**

1. Did you disclose your participation in this study to family members/significant others? How did you disclose, at what point? If not what was the reason?
2. For those who disclosed their participation to family members/significant others, how did you explain the study to them? How did they react?
3. Were there any concerns raised by family members/significant others regarding your participation in the study? What are some of these issues/concerns? ***(Probe for risks, fears, health concerns, absence from home, absence from work and others. Is this experienced differently for female and male participants?)***
4. What reasons/explanations did you give to them as to why you wanted to join the study?
5. Who amongst family members/significant others was involved in or influenced your final decision? Why them? Anyone else? ***(Is this what normally happens when making decisions?)***
6. For those who did not get support from family/significant others for participating in the study, what implications did participating have on your relationships with family, relatives and friends? ***(Is this experienced differently for female and male participants?)***

**c) Cost of participating in the study (30 min)**

1. Thinking about yourself and others who have participated, what factors do you think had to be weighed/considered when making the decision to participate in this study? ***(Probe about time, procedures, compensation, safety concerns, anything else? Is this experienced differently for female and male participants?)***
2. What factors made it easier for one to make the decision to participate in the study? ***(Probe for both study related and family/social factor; is this experienced differently for female and male participants?)***
3. What factors made it difficult to make the decision to participate in the study? ***(Probe for both study related and family/social factors; is this experienced differently for female and male participants?)***
4. What aspects of your life did you think were going to be most affected by your participation in this study and how? ***(Probe about marital, other family/social, work or business related issues)***
5. Being a participant now, what implications do you think this kind of study has on the participants’ lives? ***(Probe for before, during and after the study: Family relations e.g. child care? Other social relations? Work/business? Any other responsibilities? Is this experienced differently for female and male participants?)***

**D. CONCEPTS OF THE CHALLENGE STUDY**

1. As you are aware, this study required that participants are injected with the malaria parasite, observed and then treated with anti-malarials once they develop the malaria.
2. What do you personally think of the idea of infecting people with pathogens in research? ***(probe for pros and cons, and how community might feel/understand this concept?***)
3. If the research centre is to continue with this kind of research, what might be some of the issues? Why might these arise? How can these issues be addressed?
4. What might be the risks of directly infecting people with pathogens? What can be done about these risks?
5. Whose role/responsibility is to ensure that the research is safe for participants? Why do you say so?
6. On what types of diseases can research of this nature (where people are deliberately infected with the diseases-causing organs) be done? What makes you say so? Would you be able to participate in or not participate in? (Pneumonia, cholera?)
7. Generally, what do you think about the idea of injecting healthy people with malaria parasites and observing the outcome? ***(Assess for perception of risk involved)***
8. What are the concerns regarding this type of study from the community?

**E. FUTURE STUDIES**

1. Would you be willing to participate in a similar study (challenge) in the future?
2. How would you feel about a family member, relative or friend participating in this kind of study? What are your reasons?
3. If anything was to change about how the study is conducted, what would it be?
