# Supplementary material for: Deliberately infecting healthy volunteers with malaria parasites: Perceptions and experiences of participants and other stakeholders in a Kenyan‐based malaria infection study
Source: Bioethics. 2020 Jul 9;34(8):819–32. doi: 10.1111/bioe.12781 (PMC7689838; doi:10.1111/bioe.12781)
Supplement: Supplementary file 2 — Appendix 2 [file BIOE-34-819-s002.docx]

**IN-DEPTH INTERVIEWS/FOCUS GROUP DISCUSSIONS (FGDS) GUIDE**

**BROAD AREAS OF EXPLORATION**

**A. PARTICIPANTS**

**1. Concept of the challenge study**

1.1. Perceptions of participants of the idea of infecting people with pathogens in research

1.2. Explore Participant’s families’ perceptions regarding the challenge study; how the participants explained the study to their significant others

**2. Informed consent process**

2.1. Adequacy of information; what more information would you have liked to have?

2.2. Comprehension of the information provided E.g. what participants think the aims of the study were? What do you think the study was about?

2.3. Provision to ask questions; participant’s perceptions regarding the ability of the study team to respond to all issues they raised

2.4. Awareness of the right to withdraw from the study at whatever point

2.4.1. Ease of exit given that treatment has to be completed before leaving the inpatient settings

**3. Experiences of participating in the study**

3.1. Positive experiences of participating in the study

3.2. Negative/unpleasant experiences of participating in the study

3.3. From their experience would the participants do it again? /recommend a family member or friend to the study; reasons why?

**4. Motivations for participating**

4.1. Reasons why people participate in such challenge studies; participants’ reasons for participating

4.2. What factors did you consider before deciding to take part in the study? (Risk assessment)

4.3. Did the participants at any point in time feel like they wanted to withdraw? Reasons for staying? Reasons for wanting to withdraw?

4.4. Of all the activities and procedures in the trial which ones were the most unpleasant/difficult?

**5. In-patient settings**

5.1. Knowledge regarding reasons for having to be resident at the selected venue

5.2. Experience of staying at the selected venue

5.3. Challenges experienced during the stay; dislikes

5.4. What worked well during this time; any benefits of being resident

5.5. Concerns raised by family members regarding participation in the Malaria challenge study; about having to stay at the challenge facility

**6. Relationships with the Clinical/Trial Staff**

6.1. Ability to respond to issues raised by the participants

6.2. Friendliness/approachable

6.3. Timeliness of response to issues

**7. Potential to participate in future studies**

7.1. Would participants be willing to participate again in similar studies in the future? Why?

7.2. If the levels of benefits were lower (in a similar study), would there still be a willingness to participate why/why not?

7.3. Willingness to participate in a different type of study (not similar to challenge study) with lower levels of benefits

**B. CLINICAL TRIAL TEAM/FIELDWORKERS**

**8. Concept of the challenge study**

8.1. Perceptions of other stakeholders of the idea of infecting people with pathogens in research

8.2. Any perceived boundaries as to the type of studies that can be done using this method

8.3. Any concerns regarding this type of study

**9. Informed consent process**

9.1. Issues regarding the study that are most challenging to discuss?

9.2. What are some of the issues being raised by participants during the consenting process; what are the main concerns

9.3. What aspects of the trial are participants having most difficulty with? What aspects seem to be the most challenging to comprehend

**10. Relationships between the Clinical/Trial Staff and the participants**

10.1. Whether participants are asking questions/raising any issues with them

10.2. Efforts being made to make participants feel that they can approach the trial staff

10.3. Any issues that you have not been able to deal with? Trial related/ Non-trial related;

**C. COMMUNITY REPRESENTATIVES**

11. **Concept of the challenge study** (Give brief introduction regarding challenge studies and their aims)

11.1. Perceptions of community members of the idea of infecting people with pathogens in research

11.2. Any perceived boundaries as to the type of studies that can be done using this method

11.3. Community’s perceptions regarding this type of studies

11.4. Perceived concerns regarding participation in the malaria challenge study

**12. Community Engagement**

12.1. What information regarding this type of study should be given to the community members?

12.2. What groups/categories of people in the community should information about this study go to?

12.3. Any perceived concerns regarding the participants having to stay at in-patient settings for the duration of the study; other issues that families of participants might raise regarding the study

**13. Motivations for participating**

13.1. Reasons why people participate in such challenge studies

13.2. What factors do you think motivate people to participate in this type of study?

13.3. Any concerns/comments regarding the level of benefits in this type of study; appropriateness of levels of benefit
