# Supplementary material for: Deliberately infecting healthy volunteers with malaria parasites: Perceptions and experiences of participants and other stakeholders in a Kenyan‐based malaria infection study
Source: Bioethics. 2020 Jul 9;34(8):819–32. doi: 10.1111/bioe.12781 (PMC7689838; doi:10.1111/bioe.12781)
Supplement: Supplementary file 3 — Appendix 3 [file BIOE-34-819-s003.docx]

**MALARIA CHALLENGE COHORT 111 OBSERVATIONS CHECKLIST**

**OBJECTIVES:**

- To observe and document screening and consenting processes for the CHMI study, in order to understand contextual considerations for this study, and help refine subsequent data collection methods and tools.
- To observe and document verbal and non-verbal communication during the screening and consenting processes.

# A. GENERAL INFORMATION

Date: __/__/2018

Venue: IAVI grounds Observer: Irene Jao

Type of activity: CHMI cohort 111 re-screening and re-consenting exercise (Day __)

Time scheduled to start: _________ Time started: ___________ Time ended: ___________

Comments re start/end times: _____________________________________________________

_____________________________________________________________________________

KEMRI staff in attendance (total): Men______ Women______ (Roles) _________________

_____________________________________________________________________________

Attendance: At start: Men________ Women________ At end: Men________ Women_______

Already screened before: Men________ Women________

No. not finished screening: Men________ Women________

Reasons:

1. Opted out: Men________ Women________
2. Participated before: Men________ Women________
3. Over age: Men________ Women________

Other reasons:

1. _____________________________________________ Men________ Women________
2. _____________________________________________ Men________ Women________
3. _____________________________________________ Men________ Women________

______________________________________________________________________________

______________________________________________________________________________

# INFORMATION GIVING PROCESS DURING THE SESSION

- Describe the information giving approach used i.e. *(e.g. read whole sheet; or summarized sheet), and then add any extra info given.*
- Who gave information (Clinician, other staff etc.) and at what point?
- How long did the information giving process last?
- Were there opportunities of Q/A during the discussions?
- Were any copies of information handed over during/after the discussion? If so, what was given, to who and what was the content?
- Was the process smooth flowing/had any difficulties? If the latter was the case, at what point and what was the cause?
- Overall what seemed to work well/and what did not work well during the information giving process.
- How did the team interact/relate during the process among themselves, with community members; and any other?

1. **COMMUNITY REACTION TO THE INFORMATION**

- What was the mood of the group (any indication why)?

1. At the beginning of the meeting, describe briefly:
2. At the end of the meeting, describe briefly:

- Did everyone contribute to the discussion? Anyone dominating; anyone unengaged; any sense of why in either case? Which individuals?
- How did people react to information over the course of the meeting; verbally, non-verbally? Indicate (age/gender/status), who’s saying/asking what.
  1. Which information captured people’s attention the most? Why/in what way e.g. amusement, irritation, raised expectations, concerns?
  2. Which information were people least interested in? Why/in what way?
  3. Which topics if any caused disagreement amongst people attending the meeting and who (which groups/individuals) disagreed, what was this based on and how was it dealt with?
  4. Which explanations/information seemed to provide most reassurance (if applicable), and why?
- Were there any expectations raised by community members? To who were these concerns/expectations raised and how were these addressed?

**ADDITIONAL OBSERVATIONS:**

- Any concerns about safety?
- Issues around future benefits?
- Any community members who opted out?
- Any comments about those who did not continue with the screening/consenting process (What issues were raised?)

**NB: Please capture information on informal discussions held pre and post meetings with community members, and KEMRI staff.**

**QUESTIONS AND ANSWER SESSIONS**

| **Questions** | **At what stage of the discussion** | **What, who, why? (gender, age)** | **Response from team;**  **verbal/non-verbal during meeting** | **Pending/follow-up issues** |
| --- | --- | --- | --- | --- |
|  |  |  |  |  |
