# Supplementary material for: Deliberately infecting healthy volunteers with malaria parasites: Perceptions and experiences of participants and other stakeholders in a Kenyan‐based malaria infection study
Source: Bioethics. 2020 Jul 9;34(8):819–32. doi: 10.1111/bioe.12781 (PMC7689838; doi:10.1111/bioe.12781)
Supplement: Supplementary file 4 — Appendix 4 [file BIOE-34-819-s004.docx]

1. INFORMATION GIVING AND CONSENTING PROCESS
   1. Information giving process
      1. Sources of information about CHMI study in the community
      2. Knowledge about the CHMI study and what it involves
      3. Perceptions about information-giving
      4. Perceptions about recruitment criteria
      5. Perceptions about community engagement
   2. The screening process
      1. Perceptions about screening
      2. Perceptions about being successful at screening but not recruited into the study
      3. Perceptions about screening failure
2. DECISION MAKING FOR PARTICIPATION IN CHMI
   1. Motivation for participation
      1. Altruistic reasons
      2. Availability at the time of the study
      3. Compensation
      4. Personal satisfaction
      5. How the information about the study was given
      6. Benefits of screening, knowing health status
      7. Reasons related to knowledge about KEMRI and research
      8. The study is safe
   2. Barriers to participation
      1. Distance to Kilifi as a barrier
      2. Staying away from family and daily routines
      3. Blood draws and blood volumes
      4. Discouragement from family, friends and others
      5. Safety concerns
      6. No barriers
      7. Political instability at the time of recruitment
      8. Screening as a barrier
   3. What participants weighed before decision making
   4. Perceptions about study requirements
      1. Experiences and feelings about requirement of contraceptive use
      2. Feelings about requirement for use of mosquito net Feelings about 21-day stay and confinement
      3. Feelings about 3-day treatment before leaving in-house facility
   5. Disclosure to significant others and decision making for participation
3. VOLUNTARY PARTICIPATION AND THE RIGHT TO WITHDRAW FROM CHMI STUDY
   1. Perceptions about voluntariness and right to withdraw from CHMI study
   2. Role of clinicians in participant voluntary participation and right to withdraw
4. EXPERIENCES OF PARTICIPATION IN CHMI STUDY
   1. Experience during participation in the CHMI study
      1. Positive feelings about being in the study
      2. Conduct of participants at the in-house facility
   2. Concerns, dislikes, difficulties and challenges faced in the study
5. PERCEPTIONS ON DELIBERATE INFECTION
   1. Perceptions about deliberate infection with malaria parasites
      1. Feelings about malaria challenge study
      2. Fears and concerns about deliberate infection with malaria parasites
   2. Risks involved in challenge studies
   3. Responsibility of ensuring safety of study participants
   4. Concerns if challenge studies are to continue
      1. Need for public engagement
      2. Giving back to the community
      3. Concerns that CHMI study staff could have
      4. ‘Research fatigue’
   5. Willingness to participate in future challenge studies
      1. Participants' willingness to be involved in future challenge studies
      2. Whether participants will accept family members to participate in future challenge studies
   6. Recommendations about future study procedures
      1. Separating male and female participants
      2. Feelings about having financial management talks during the study
      3. Recommendations for changes in study procedures
      4. Feelings about counselling sessions
   7. Perceptions about injection with other pathogens
6. IMPLICATIONS OF PARTICIPATION ON PARTICIPANTS' LIVES
   1. No implications
   2. Effects on social aspects of participants' lives
   3. Alternative support or arrangements made during participation
   4. Implications on future studies
   5. Financial implications of participating in CHMI study
      1. Financial benefits of participating in CHMI study
      2. Financial implications of participating in CHMI study
   6. Gender aspects in CHMI
      1. Interference with child care
      2. Effects on roles and responsibilities in the home
      3. Gender issues
   7. Implications of participation post-study
