# Supplementary material for: Deliberately infecting healthy volunteers with malaria parasites: Perceptions and experiences of participants and other stakeholders in a Kenyan‐based malaria infection study
Source: Bioethics. 2020 Jul 9;34(8):819–32. doi: 10.1111/bioe.12781 (PMC7689838; doi:10.1111/bioe.12781)
Supplement: Supplementary file 5 — Appendix 5 [file BIOE-34-819-s005.docx]

**KEMRI Wellcome Trust Research Programme: Patient Information and Consent Form**

***Study Title:*** *Controlled Human Malaria Infection (CHMI)*

***Lay Title:*** *Understanding how resistance to malaria develops in healthy volunteers by infecting then with malaria parasites.*

| Institution | Investigators |
| --- | --- |
| *KEMRI* | *Philip Bejon (PI), Faith Osier, Francis Ndungu, Kevin Marsh, Melissa Kapulu, Patricia Njuguna, Juliana Wambua, Bernhards Ogutu, Elizabeth Juma, Sam Kinyanjui, Maureen Njue, Dorcas Kamuya* |
| *Sanaria Inc* | *Stephen Hoffman, Thomas Richie, Peter Billingsley* |
| *University of Cambridge* | *Peter Bull* |
| *Pwani University* | *Osman Abdullahi, Cheryl Andisi* |

**Introduction:**

Malaria causes disease and death especially in children under the age of five. People living or who have grown up in areas where there is transmission of malaria have been exposed to the disease and over time develop resistance. These individuals, often adults, can be exposed to infection but are unable to develop disease. We need to understand how this resistance affects how malaria develops after which we then study how the body responds and how the malaria parasite in the body develops and grows. We can observe up to the point where the person develops the malaria. One way to do this is to infect healthy adults with malaria by injection. This information would help us to develop and test vaccines that can prevent disease and transmission of malaria.

**Who is carrying out this study?**

This study is being carried out by researchers from KEMRI in Kilifi in collaboration with researchers from KEMRI in Nairobi. KEMRI is a government organisation that carries out medical research to find better ways of preventing and treating illness in the future for everybody’s benefit.

**What is this study about?**

- One problem KEMRI is currently trying to learn more about is malaria. In this study, we aim to find out how resistance to malaria in healthy adults who have been previously exposed develops and understand what mechanisms lead to this resistance. Studies where healthy volunteers are infected with malaria parasites (challenge studies) have previously been conducted in healthy African adults before (adults from Kenya, Tanzania and Gabon) and in other parts of the world including adults from America, Europe and Australia. These studies have been safe and have shown that this is an effective way of determining the development of resistance to malaria. However, more research is required in individuals with different levels of past exposure to malaria to confirm the findings of previous research.
- In this study we plan to infect healthy adults with malaria and then closely examine how the body fights the malaria parasites. The malaria parasites will be injected into the vein in your arm or hand. These parasites have been developed by American researchers and have previously been given to volunteers in The Netherlands, UK. USA, Kenya, Tanzania, and Gabon. Following injection you will stay either in our clinical research facility in Nairobi or at Pwani University in Kilifi full time until you are treated for malaria. We hope that the information we get from this study will help in development of a malaria vaccine.
- We aim to screen a total of 2,000 individuals who have grown up in areas where there is malaria*.* These individuals will be tested for their previous exposure to malaria and other test to confirm if they are eligible to take part in the study.
- From these we hope that a minimum of 200 volunteers will be found to have past exposure to malaria. These will then be eligible for this study. They will be recruited into the study only if they consent.
- All volunteers infected with malaria will be followed up closely to observe if and when they develop signs and symptoms of malaria. During this time, they will have to stay in an in-patient ward (Nairobi Centre for Clinical Research (CCR)) or at Pwani University (in Kilifi) for close monitoring. When they develop sign and symptoms of malaria, they will be treated with currently approved effective anti-malarial drugs.
- We are asking your permission for you to participate in this study.

**What are the requirements for you to be involved in the trial?**

In order for you to be involved in the study, you must be:

- A healthy adult aged between 18 to 45 years.
- Able and willing (in the investigators’ opinion) to comply with all study requirements.
- Willing to stay at Pwani University near KEMRI Kilifi or KEMRI CCR Nairobi for the whole study (from day of administration of malaria parasites until completion of a course of anti-malaria therapy).

You cannot participate in the study if:

- You are intending to leave Kilifi or Nairobi between screening and completion of malaria challenge.
- You have used antibiotics which could treat malaria in the 30 days prior to involvement in the study.
- You have previously received an investigational malaria vaccine.
- You have had any blood products in the three months preceding your involvement in this trial.
- You have problems with your immune system.
- You have a red blood cell abnormality that may affect study outcome.
- You are pregnant, breast feeding or intend to become pregnant during the study.
- You have allergic disease or reactions likely to be exacerbated by malaria infection.
- You have a history of cancer.
- You have a history of a serious psychiatric condition that may affect participation in the study.
- You have any other serious chronic illnesses.
- You have injected drugs at any time in the last 5 years.
- You have HIV infection.
- You have a history of heart disease.
- Close family members have developed heart disease when aged younger than 50 years.

Mild conditions, such as childhood asthma which is well controlled would not automatically exclude you from participating. If you are unclear whether you are eligible to be involved in the study you can contact the study team who will be able to advise you.

**What will it involve for me?**

If you agree to participate, we will first need to check your health status to find out whether it is possible for you to be infected with malaria by finding out more about you and your exposure to malaria. For those from Ahero, you will be asked to travel to Ahero Clinical Trials Unit (ACTU) in Ahero where screening checks and tests of your health status and eligibility for the study will be conducted. For those from Kilifi, you will be asked to travel to KEMRI in Kilifi. For those in Nairobi, you will be asked to travel to KEMRI Centre for Clinical Research where your checks will be done.

For this you will attend a screening appointment where*:*

- A member of the clinical team will discuss the trial with you and answer any questions you have.
- A checklist will be used by the clinician to assess your understanding of the study in order for us to be confident that you fully understand what taking part will involve. You need to answer all questions correctly in order to take part in the study. You will then be asked to sign a consent form. You are free to withdraw at any time without giving a reason, but you may be asked to return to the clinic for follow up for safety reasons.
- A small amount of your blood from a vein in your hand or arm will be taken to carry out medical tests and exposure to malaria. The amount taken will be 20mls (about 2 tablespoons) of blood.
- A test of your urine will also be performed and this will include a pregnancy test for women.
- If we find you have any health problems or abnormal blood tests, we will explain this to you and refer you for any further tests or treatment you need at the most appropriate health facility. We will not ask you to continue participating in this study.
- **HIV testing**: One of these blood tests will be an HIV test. A trained counsellor will explain the HIV test to you before it is done, and discuss the results with you afterwards. All results will be kept confidential. You will be given your results in private. If you are HIV positive, you will be referred to a local health facility where you will receive standard medical care according to the Ministry of Health guidelines.
- The results of whether you are eligible to take part in the study will not be available immediately. It will take between three to one hundred and twenty days (3 to 120 days) after screening for the results to be ready depending on whether you are from Ahero, Kilifi or Nairobi. The tests to determine exposure to malaria will be done in KEMRI Kilifi and the blood drawn for these tests will need to be taken to Kilifi. Therefore, you will have to wait until you have been called by the study clinician to be told that you are eligible to participate in the study.
- After the screening is completed, you will be asked to return to your residence either in Ahero, Kilifi or Nairobi and wait to be called to find out if you are eligible to take part in the study. Once you have been called and made aware of eligibility, if you are still willing to take part in the study, you will be asked to travel either KEMRI Kilifi or KEMRI Nairobi CCR and this will be for all those eligible whether from Kilifi or Ahero and Nairobi respectively.

**If you fit the requirements of this research and are found able to receive the injection with malaria, we will:**

1. **Repeat health check a day before injection of malaria:** We will ask you to return to the respective facilities for a review, blood test and, for female volunteers, a urinary pregnancy test a day or two before the challenge to ensure you are completely healthy and can enter the malaria trial. We will also collect a 59mls (about 6 tablespoons) blood sample at this time to carry out medical tests and exposure to malaria.
2. **Injection with malaria and in-patient stay at Pwani University (for Kilifi) or study clinic (for Nairobi):** You will be given one injection of malaria parasites in the vein on your arm or hand. After this injection you will stay at Pwani University near KEMRI in Kilifi or in a clinic at KEMRI Centre for Clinical Research, Nairobi, full time until you have completed malaria treatment. You will be expected not to leave Pwani University or the clinic during this time and to sleep at Pwani University or clinic. This is important because you will need to be monitored on a daily basis and we do not want you to be exposed to getting malaria.
3. **Health checks and blood tests following injection with malaria:** After injection of the parasites, you will be reviewed at least twice a day by the study staff. But if you feel unwell then you will be reviewed more often. A nurse or doctor will be available 24 hours a day in the clinic or at Pwani University so there will always be someone to look after you. Days 1-6 following injection of malaria parasites only one blood sample will be taken on day 5 of 32mls (about 3 tablespoons). On days 7-14 following injection of malaria parasites, a blood test will be taken twice a day in the morning between 7am and 10am and in the late afternoon between 3pm and 7pm. The total amount of blood taken during this time will vary but will be between 4ml and 38mls (a few drops and 4 tablespoons). On days 15-21 following injection of malaria parasites, a blood test will be taken once a day in the morning between 7am and 10am and the amount taken will vary between 4ml and 41mls (a few drops and 4 tablespoons).

- As soon as you are diagnosed with malaria you will be started on a course of anti-malaria treatment. Once you are treated and found to have your malaria infection cleared, you will be able to go back to your home. This will be for up to 24, 48, and 72 hours after starting anti-malaria treatment and a blood test will be done to make sure the malaria infection has been treated. In addition a blood test (1ml, a few drops) will be taken at 72 hours after starting treatment to check your health status after treatment.
- If you become unwell with malaria during the course of the challenge study then you may be admitted to a Hospital in Kilifi, Mombasa or Nairobi as a precaution until you have recovered, but it is very unlikely that this will be necessary. The hospitals you may be admitted to include: Kilifi – Kilifi County Hospital; Mombasa – Mombasa Hospital, Aga Khan Hospital; and Nairobi – Nairobi Hospital, Aga Khan University Hospital and Mater Hospital. We anticipate that you will be diagnosed with malaria between 9-14 days after injection of malaria parasites. In the event that we do not diagnose you by day 21 after injection of parasites then we will give you a course of anti-malarial treatment anyway so that any parasites that we have not detected will be killed.
- As long as you are well and there are no malaria parasites in these blood tests you can leave Pwani University or clinic 72 hours after starting malaria treatment. So the longest time you could spend in the clinic or Pwani University is approximately 24 days.

1. **Follow-up visits:** We will ask you to return to the facility in Kilifi or the clinic in Nairobi for a health check 35 days after injection of malaria parasites. We will take a blood test at these visits of 55mls (approximately 5 and a half tablespoons). In case you feel unwell while at home you should contact the study team or come for review.

**What amount of blood will be taken during the study?**

The total amount of blood taken during the study will be 412mls (about 42 tablespoons) over approximately 3 months. These blood tests are to assess your health status and immune response to malaria. The amount taken at each visit will vary between 4mls and 55mls (a few drops and 5 and a half tablespoons). This amount of blood should not cause any problems in healthy people. We will give you a copy of your blood tests if you request them but we will inform you about the results. The amount of blood taken at each visit is outlined in the table below:

| Schedule | Screening visit | Day before injection | Days 5-14 after injection | Days 15-24 after injection | Day 35 after injection |
| --- | --- | --- | --- | --- | --- |
| Blood volume per visit (ml) | 20 | 63 | 4-38 | 4-41 | 55 |
| Blood volume per visit (tablespoons) | 2 | 6 | 2/5 - 4 | 2/5 - 4 | 5 |
| Cumulative blood volume (ml) | 20 | 83 | 275 | 357 | 412 |

**Considerations before taking part in the study:**

1. **Medications:** You should not take any drugs other than vitamin pills, contraceptive pills or those medications assessed as appropriately safe during a malaria challenge by the doctor at screening. This also applies for drugs bought over the counter. Of course, your health and well-being is much more important than the conduct of this study and if at any time you need any medication then you should take it. However, it is very important that you **let us know before you start on any treatment**. For example, any antibiotics that you take within 4 weeks of the planned challenge day may affect the malaria parasite and mean that we cannot include you in the study.
2. **Exposure to mosquito bites:** We will test your blood for malaria parasites at your screening visit as there is need to be no malaria parasites in your blood for you to be in the study. It is very important that you do not become infected with malaria between your screening visit and the day of injection of malaria parasites. For those living in Ahero and Kilifi, you will need to sleep under bed nets to prevent exposure to mosquito bites. These will be provided to you during the screening visit. For those in Nairobi, while it is not possible to catch malaria in Nairobi, it is possible to catch malaria in many other parts of Kenya. We therefore ask you not to leave Nairobi between your screening visit and day of injection with malaria parasites.
3. **Pregnancy:** Malaria infection can be particularly dangerous during pregnancy to both the mother and the baby. Women are therefore asked to use an effective method of contraception such as pills or condoms during the study period to avoid pregnancy. If you are not already using any contraception and require it, you will be referred to the nearest family planning clinic to obtain them. A pregnancy test will be carried out at screening, before the injection with malaria parasites and again before anti-malarial treatment is started.
4. **Blood Donation:** You should not donate blood whilst you are taking part in this study.

**Are there any risks or disadvantages to me of taking part?**

Our priority for every participant is their well-being. However there are some potential risks from taking part in the study.

- **Blood sampling:** There may be some pain and bruising associated with blood drawing which will resolve after a few days. There is a small risk of infection. This risk is minimized by use of pre-packaged sterile equipment and trained staff.
- **Injection of malaria parasites:** You may experience some injection site pain, redness or warmth where the parasites are injected however this is most likely to be mild. With any injection there is a risk of a severe allergic side effect. These include skin swelling, shortness of breath and light-headedness or fainting. Medical equipment necessary to treat serious reactions will be available. You will be closely monitored by a study doctor who will deal with any injection related illness should they occur.
- **Malaria infection:** Malaria is a serious disease and can lead to death if left untreated. Most people do not develop symptoms but have the parasites in their blood, whilst others only develop a mild form of the disease, and others may become very seriously sick and may die if they are not treated. Therefore, monitoring will be done throughout the time from challenge to ensure that they are treated and the infection is cleared before they are released from the study. The risks of taking part in this study are very low providing that you stay at Pwani University or clinic after injection of malaria parasites so that the study team can look after you. However, if untreated, the malaria infection that we propose to give you could result in death. Worldwide over 1,300 people have been deliberately infected with malaria in challenge studies to date and all have made a complete recovery.
- We intend to diagnose and treat your malaria infection before the onset of symptoms however it is likely that you will develop some symptoms of malaria, such as a flu-like illness, fever, chills, headache, muscle pains, diarrhoea or vomiting. These symptoms may be severe enough that you may need to be in bed for a couple of days. In addition to treating you for the malaria, we will prescribe pain-killers such as paracetamol if required. Symptoms can start or persist after treatment has started but usually last no more than 1 to 3 days.
- If malaria is not properly treated, possible complications include jaundice, kidney failure, fluid in the lung, low blood sugar and collapse. Seizures, altered consciousness, coma and even death may occur. It is therefore very important you stay in the inpatient clinic once we have given you the injection of malaria parasites and that if you want to leave the study you allow us to treat you first.
- For 6 months after the injection of malaria parasites you should contact the study team if you develop any of the symptoms of malaria as detailed above.
- The malaria parasites used in this study are known to be very sensitive to the drugs that will be used in the treatment of infection and possible infection. The drugs are all recommended for the treatment of malaria. We therefore do not expect any treatment failures as this has not happened in previous studies.
- **Treatment of malaria:** one of the drugs you will be treated with is called artemether-lumefantrine. Arthemeter-lumefantrine is a combination drug consisting of artemether 20mg and lumefantrine 120mg per tablet. A treatment course consists of 6 doses of 24 tablets. The first 4 tablets will be given when you are diagnosed with malaria followed by dose after 8 hours then a dose every 12 hours for 2 days (4 tablets each time). Arthemeter-lumefantrine is a recommended treatment for malaria. The infecting parasites are known to be killed by Arthemeter-lumefantrine and this drug is the current treatment for malaria in Kenya. We will need to watch you take each of these doses and will continue taking blood to look for parasites until the blood tests are negative for malaria parasites. In case you may not be cured with Arthemeter-lumefantrine you will be treated with either chloroquine or Sulphadoxine/pyrimethamine (Fansidar) which is also recommended treatment for malaria in Kenya.
- Like all medicines, Arthemeter-lumefantrine can cause side effects, although not everybody gets them. Most side effects reported have been mild and have not lasted very long. Side effects include Headache, dizziness, loss of appetite, weakness, fever, chills, tiredness, muscle/joint pain, nausea, vomiting, abdominal pain, cough, and trouble sleeping may occur. Serious side effects that may occur are chest pain, severe dizziness, fainting, and fast or irregular heartbeat. Many people using this medication do not have serious side effects. A very serious allergic reaction to this drug is rare. Signs of severe allergic reactions include rash, itching or swelling especially of the face, tongue or throat, severe dizziness, and trouble breathing.
- Allergy to the components of Arthemeter-lumefantrine may prevent you being treated with Arthemeter-lumefantrine. Please let us know if you think this might apply to you. If need to stop taking Arthemeter-lumefantrine during the study then you will be treated with another effective medication.
- Paracetamol may be given to you to reduce fever, muscle and joint pain, back ache and headache. If you get any side effects from the malaria medicine such as itching, nausea or vomiting, you will be given another medicine called cyclizine to help relieve those symptoms.
- Other potential complications to be aware: In 2008, a 20-year-old healthy female volunteer in the Netherlands taking part in a malaria vaccine study developed chest pain two days after finishing treatment for malaria. She was admitted to hospital but was discharged home well with no long-term side effects. We do not know whether the event was related to her malaria infection (given by mosquito bite), the vaccine that she received, the medicine used to treat malaria (Malarone®) or something else.
- In February 2013, a 23-year-old healthy male volunteer also in the Netherlands taking part in a malaria vaccine study (of a different malaria vaccine) developed chest pain and a heavy feeling in his left arm. After further evaluation he was diagnosed with a condition called “myocarditis,” which is an inflammation of the heart muscle. We do not know whether this illness was related to malaria infection (given by mosquito bite), the vaccine that he received, the medicine used to treat malaria (Malarone®), a viral infection unrelated to the study or something else. The myocarditis resolved without treatment.
- An independent committee will monitor this research continuously to ensure participants safety and rights are respected at all times.
- If for any reason the doctors looking after you think you would benefit from leaving this trial, they will recommend this and ensure that you receive the normal treatment given to people who are not in the trial.
- **Time and travel:** This study will involve your time and you may have travel costs. However we will compensate for your time and travel expenses. We will reimburse the amount you spend on fares for each journey you make to the health facility for research related visit. The amount reimbursed will depend on the amount of money spent for travel for each participant and this will vary whether you are from Ahero, Kilifi or Nairobi. In addition for reimbursement of transport fares, we will compensate you for time or wages lost on that day at a rate of Ksh500 for each time you come to the clinic. For the duration that you will stay at Pwani University or health facility during the study, we will compensate for your time, lost earnings, and inconveniences at a rate of Ksh2, 000 per overnight stay. In addition, the study team will cover all related costs including accommodation and meals. These rates are based on recommended guidelines for daily wages and out of pocket allowance for overnight stay at KEMRI.

**Are there any advantages to me of taking part?**

- If you participate in this study, you will receive medical care for any acute ailments from the day of injection of malaria parasites until the completion of the study free of charge.
- Treatment of chronic illnesses or long term injuries unrelated to the study procedures will not be paid for by the study. If you are found to have such illnesses/injuries, you will be treated under the existing government programs.
- By participating in this study, you will be helping with malaria research that may help development of a malaria vaccine that would bring health benefits to future generations of children.

**What happens if I refuse to participate?**

- All participation in research is voluntary.
- You are free to decide if you want to take part. You will still receive the recommended standard of care if you do not take part.
- If you do agree you can change your mind at any time and withdraw from the research. This will not affect your care now or in the future.
- However, if you decide to withdraw from the study after we have given you an injection of malaria parasites then you must agree to take a course of anti-malaria therapy.
- In the event that you leave the study before its completion, we still encourage you to have the examinations and blood tests to assess safety following injection of malaria parasites. This will involve clinic visits at 35 and 90 days following injection of malaria parasites

**What happens to the samples?**

- All samples will be coded with a unique study identification number and no personal information such as your name or date of birth will be included. Individual names are removed from all samples and replaced by codes, to ensure that samples can only be linked to the participants by people closely concerned with the research.
- Most of the research tests that will be done on the sample will be done in KEMRI either in Kilifi or Nairobi. However for some test that cannot be done in Kenya, part of the samples will be sent to laboratories overseas in Oxford and Cambridge, UK; Sanaria and University of Maryland, USA; Radboud University Nijmegen Medical Centre, The Netherlands. All the institutions that samples will go to are collaborators involved in this study and development of a malaria vaccine.
- After the research, a small portion of the samples will be stored in Kilifi for 10 years. In the future, new research about malaria may be done on these samples.
- Any future research will first be approved by KEMRI Scientific and Ethics Unit committee to ensure participants’ safety and rights are respected.
- If you decide that you don’t want to take part in the study after a sample has been taken, the sample will not be used in any of the study procedures.

**Will any genetic tests be done?**

- Some blood will be used to look at DNA in your body that makes your body respond to the malaria infection. These DNA are usually in groups called genes and will test for those that are usually responsible for immune response to malaria. This will help us better understand how the immune responses to infection are produced.
- In Kenya some people have a condition of the blood called sickle cell which causes anemia and other problems. Although this is rare, we will test your blood for this condition and if sickle cell disease is found we will let you know and refer you to a health facility. We will not automatically return results to you if there is no evidence of a problem but the results will be available to you if you request them.

**Who will have access to information about me in this research?**

- All our research records are stored securely in locked cabinets and password protected computers.
- Only the people who are closely concerned with the research will be able to view information from participants, in order to be sure that the study is being run correctly and the health of every participant is protected. These individuals will keep the information confidential.
- In future, information collected or generated during this study may be used to support new research by other researchers in Kenya and other countries on malaria and other health problems. In all cases, we will only share information with other researchers in ways that do not reveal individual participants’ identities. For example, we will remove information that could identify people, such as their names and where they live, and replace this information with number codes. Any future research using information from this study must first be approved by a local or national expert committee to make sure that the interests of participants and their communities are protected.
- Study information will be made available through an open repository. The information to be made available will be anonymized so that there is no link to participants and will include data on antibody responses, parasite growth rates and any other data generated from samples obtained in this study, both generated from this current protocol or any future studies which will require additional ethical approval.
- The information that might be shared includes information on inherited factors in individual participants. Any future research using information from this study must first be approved by a local or national expert committee to make sure that the interests of participants, their families and the community are protected.
- For data which includes human genetic or genomic data, the information that might be shared includes information on inherited factors in individual participants. Any future research using information from this study must first be approved by a local or national expert committee to make sure that the interests of participants, their families and the community are protected.
- In order to do this study, we will share anonymized individual and summary information we collect or generate with Sanaria Inc., University of Oxford, University of Cambridge, and RUNMC, in ways that do not reveal individual participants’ identities.

**Who has approved this research?**

All research at KEMRI has to be approved before it begins by the KEMRI Scientific and Ethics Review Unit and the Oxford Tropical Research Ethics and the Pwani University ethics review committees who look carefully at planned work. They must agree that the research is important, relevant to Kenya and follows nationally and internationally agreed research guidelines. This includes ensuring that all participants’ safety and rights are respected.

**What if I have any questions?**

**You are free to ask questions of any staff at any time. You can also contact the research team using these contacts:**

Prof. Philip Bejon, KEMRI Wellcome Trust Research Programme, P.O. Box 230, Kilifi. Telephone: 0709983549 or 0722 203417, 041 7522063

Dr. Bernhards Ogutu, CCR-KEMRI, P. O. Box 2254 – 0202, Nairobi. Telephone: 0733812613

***If you want to ask someone independent about this research please contact:***

Community Liaison Manager, KEMRI Wellcome Trust Research Programme, P.O. Box 230, Kilifi. Telephone: 0723 342 780 or 041 7522 063

***And***

The Secretary, KEMRI Science and Ethics Review Unit (SERU), P. O. BOX 54840-00200, Nairobi, Tel number: 020 272 2541 Mobile: 0722 205 901 or 0733 400 003

*This research is supported by University of Oxford who will pay for any treatment or compensation in the unlikely event of any injury resulting from the study.*

**KEMRI Wellcome Trust Research Programme consent form for**

**Controlled Human Malaria Infection (CHMI)**

***Lay Title: Understanding how resistance to malaria develops in healthy volunteers by infecting then with malaria parasites.***

I, ____________________________________ have had the research explained to me. I have understood all that has been read/explained and had my questions answered satisfactorily.

**Yes** *please tick* **I agree to participate in this research**

**Yes** *please tick* **I agree to samples being stored and used for future research**

**Yes** *please tick* **I agree to samples being exported to Oxford and Cambridge, UK; Sanaria, USA; Nijmegen, The Netherlands.**

I understand that I can change my mind at any stage and it will not affect to me in any way.

**Subject signature**: ____________________________________**Date** ___________

**Subject name:** _____________________________________**Time** ____________

-----------------------------------------------------------------------------------------------------------------

I attest that the information concerning this research was accurately explained to and apparently understood by the subject and that informed consent was freely given by the subject.

**Witness’ signature:** _____________________________________ **Date** _____________

**Witness’ name**: _____________________________________ **Time** ______________

-----------------------------------------------------------------------------------------------------------------

I have followed the study SOP to obtain consent from the____________________________________. S/he apparently understood the nature and the purpose of the study and consents to the participation in the study. S/he has been given opportunity to ask questions which have been answered satisfactorily.

**Designee/investigator’s signature:** ____________________________ **Date** ____________

**Designee/investigator’s name:**  _____________________________ **Time** ____________
